# Large-scale computations on histology images reveal grade-differentiating parameters for breast cancer

**DOI:** 10.1186/1471-2342-6-14

**Published:** 2006-10-27

**Authors:** Sokol Petushi, Fernando U Garcia, Marian M Haber, Constantine Katsinis, Aydin Tozeren

**Affiliations:** 1 School of Biomedical Engineering, Science & Health Systems, Drexel University, 3141 Chestnut St., Philadelphia, PA 19104, USA; 2 Department of Pathology, Drexel University College of Medicine, 245N 15^th ^St., Philadelphia, PA 19102, USA; 3 Godwin College of Professional Studies, Drexel University, 3001 Market St., Philadelphia, PA 19104, USA

## Abstract

**Background:**

Tumor classification is inexact and largely dependent on the qualitative pathological examination of the images of the tumor tissue slides. In this study, our aim was to develop an automated computational method to classify Hematoxylin and Eosin (H&E) stained tissue sections based on cancer tissue texture features.

**Methods:**

Image processing of histology slide images was used to detect and identify adipose tissue, extracellular matrix, morphologically distinct cell nuclei types, and the tubular architecture. The texture parameters derived from image analysis were then applied to classify images in a supervised classification scheme using histologic grade of a testing set as guidance.

**Results:**

The histologic grade assigned by pathologists to invasive breast carcinoma images strongly correlated with both the presence and extent of cell nuclei with dispersed chromatin and the architecture, specifically the extent of presence of tubular cross sections. The two parameters that differentiated tumor grade found in this study were (1) the number density of cell nuclei with dispersed chromatin and (2) the number density of tubular cross sections identified through image processing as white blobs that were surrounded by a continuous string of cell nuclei. Classification based on subdivisions of a whole slide image containing a high concentration of cancer cell nuclei consistently agreed with the grade classification of the entire slide.

**Conclusion:**

The automated image analysis and classification presented in this study demonstrate the feasibility of developing clinically relevant classification of histology images based on micro- texture. This method provides pathologists an invaluable quantitative tool for evaluation of the components of the Nottingham system for breast tumor grading and avoid intra-observer variability thus increasing the consistency of the decision-making process.

## Background

This article presents a clinically relevant classification of Hematoxylin and Eosin (H&E) histology slides based on automated image processing, supervised learning, and large-scale microtexture computations. The H&E stain dyes DNA-rich cell nuclei blue and collagen-rich extracellular matrix (ECM) pink, allowing differentiation of DNA-containing nuclei from the surrounding ECM [1]. Currently used breast tumor grading systems assess nuclear features, tubule formation, and mitotic rate to formulate a tumor grade [1,2]. Pathologists evaluate each of these parameters in small sample regions of the microscopic image and give a score of 1 to 3 in increasing order from best to worse-case scenario. The breast tumor grade is the sum of the three scores [3]. The lowest possible score (1 + 1 + 1 = 3) along with scores 4 and 5 correspond to grade I tumors. These low-grade tumors possess well-differentiated cells with low mitotic rates, and a tubular growth pattern. Intermediate grade tumors (Grade II) have a total score of 6 or 7 whereas high-grade tumors (Grade III) have a total score of 8 or 9. High-grade tumors known as poorly differentiated carcinomas, are characterized by infiltrating breast cancer with less than 10% of the lesion arranged as tubules, highly pleomorphic nuclei and many mitoses.

Pathologist-based evaluation of tissue slides for tumor grading is considered the gold standard for tissue neoplasm assessment. However, it is subject both to observer variation and variability based on the spatial focus of observation [4-7] Moreover, tumor classification based on qualitative analysis of morphology, in individual cases, is not necessarily predictive of clinical outcome [3]. Some of the patients in the 'better' prognosis category will manifest aggressive disease and vice versa. The outcome is patient mismanagement with chemo- and hormone therapy given unnecessarily to some and not provided to others who might benefit. The inconsistency between image-based grading and clinical outcome has led to studies for better markers to predict biologic behavior; these include potential development of global gene expression and genome-wide signatures for various cancers and subtypes [8-11]. In parallel, other studies have focused on automated image processing for better accuracy in tumor grading [12,13]. Hybrid segmentation methods have been used to detect nuclei from images of histology slides stained under different conditions [12-14]. An image morphometric method of nuclear grading based on Z-scoring has been developed by Bacus et al. [15] for breast Ductal Carcinoma in Situ (DCIS). Similarly, Hoque et al. [16] quantified the mean nuclear features such as area, eccentricity, elongation and compactness in recurrent and non-recurrent DCIS and determined those nuclear features that were predictive of grade and/or survival time. Wolberg at al. [17] investigated the effectiveness of a computer-based nuclear morphology evaluation technique for breast cancer prognosis and showed that nuclear morphology evaluation was a better prognosticator of disease free survival compared with lymph node status.

Our study expands the previous work as it applies large-scale computations and machine-learning algorithms that can aid in the development of new indices based on tissue micro-texture motives for classifying breast histology images. This study utilizes our previously published method of hybrid segmentation and supervised learning to identify micro-textures that can potentially be used as features to classify histology images [18]. Tissue image objects thus identified as cell nuclei by hybrid hierarchical segmentation were classified by supervised learning into three morphology categories and a category of false detection. The spatial positions of millions of cell nuclei and their morphology types were identified on histology images. The present study begins with gathering the spatial nuclei distribution information on breast histology slide images and identifies tumor regions with high nuclei density. Tubular cross sections in the images and other spatial texture parameters such as the mean distance between neighbouring nuclei were detected using the spatial information on cell nuclei distribution. Classification algorithms based on supervised learning were then used in order to classify histology section images based on morphology and texture patterns. Our results indicate that the number density of dispersed chromatin cell nuclei and the number density of breast tubular cross sections function as grade differentiating features. In combination with other texture/biomarker features derived from platforms such as, biomarker-decorated image data, global gene expression profiles, and chromosome aberration measurements, the differentiation potential of these new findings will increase due to increasing length of outcome specific feature vectors used in classification.

## Methods

### Images used for breast cancer cell identification and grade classification

The histology image dataset used for clinically relevant classification of histology images consisted of 24 H&E stained slide images. These images contained cross sections of millions of cancer cells and thousands of breast duct tubules. Each histology image in the dataset belonged to a different patient case. These cases were chosen from a large dataset associated with the breast cancer databank of the Drexel University College of Medicine (DUCOM). This database is an archive of approximately 2200 paraffin-embedded breast cancer specimens collected from 1997 to the present. The data collection and study for this project was covered under the IRB (Institutional Review Board) with study #: 10128 and protocol title: 3-D Reconstruction of Breast Carcinoma". The IRB was approved as an exempt research #4 as this project involved collection and study of deidentified existing pathological specimens (paraffin-fixed tissue blocks and histology slides). The dataset was filtered to show only lumpectomy cases and a subset was chosen to represent the three breast tumor grades (I, II, III) in the dataset at equal numbers. Two experienced pathologists who specialize in breast tumor histopathology (Drs. Fernando U. Garcia and Marian M. Haber) re-scored the histology slides of this subset in sequential order. The first eight cases from each tumor grade, identically scored from both pathologists, were used in this research. The Nottingham grading system [2,3] was used in assigning grade to histology slides. Table 1 shows the individual components of the Nottingham scoring system and the overall grade assigned unanimously by the two pathologists to the 24 whole section slide images used in the study.

**Table 1:** Nottingham scores of selected cases as graded by the pathologist experts

Study Case	Tubule Formation	Nuclear Pleomorphism	Mitotic Count	Total Score	Histologic Grade
1	1	1	1	3	1
2	1	2	1	4	1
3	1	2	1	4	1
4	1	1	1	3	1
5	2	1	2	5	1
6	1	1	1	3	1
7	3	1	1	5	1
8	1	1	1	3	1
9	3	2	1	6	2
10	2	2	2	6	2
11	2	3	1	6	2
12	2	3	2	7	2
13	2	2	2	6	2
14	3	2	1	6	2
15	2	2	2	6	2
16	1	3	3	7	2
17	2	3	3	8	3
18	3	3	2	8	3
19	3	2	3	8	3
20	3	2	3	8	3
21	3	3	3	9	3
22	2	3	3	8	3
23	3	3	3	9	3
24	2	3	3	8	3

tubular formation (TF), nuclear pleomorphism (NP), mitotic count (MC), total score, and histologic grade (HG).

**Figure 1:**
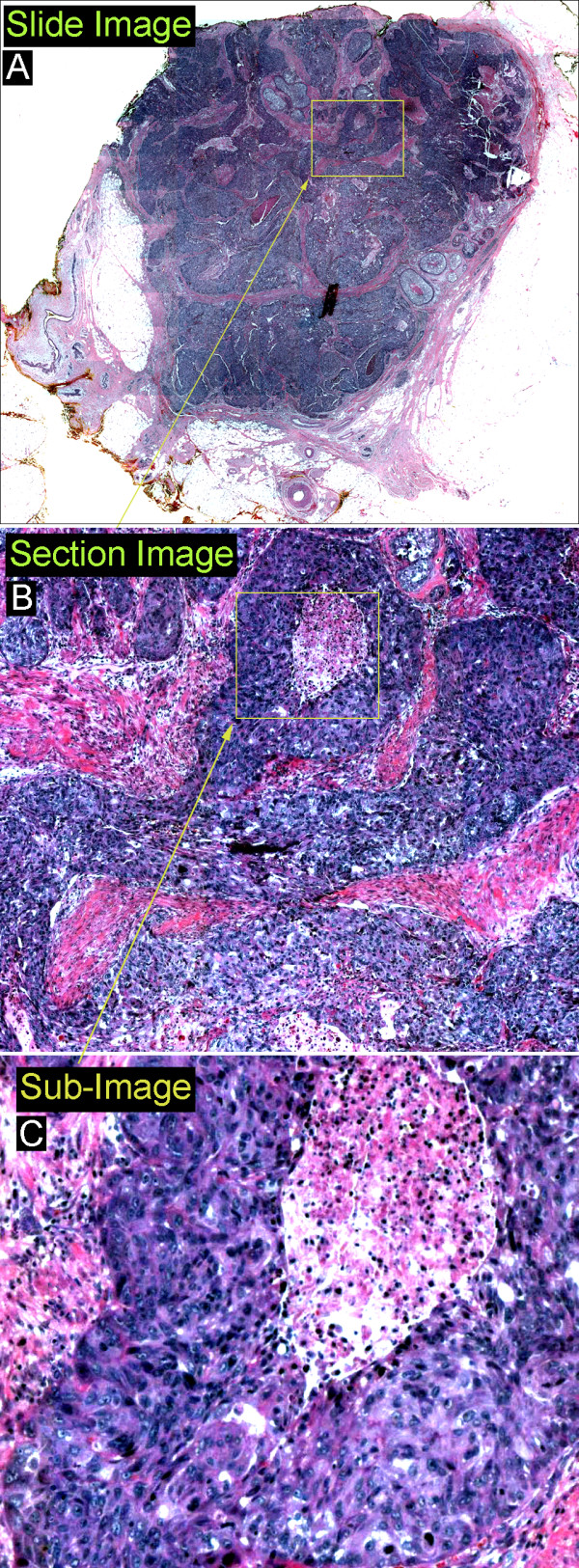
**Image dataset composition**. A: A whole section image of an invasive breast tumor. Size: 12320 × 9200 pixels (8994 um × 6716 um), B: Section image (1/64 of slide image). Size: 1540 × 1150 pixels (1124 um × 840 um), C: Sub-image (1/25 of section image). Size: 308 × 230 pixels (225 um × 168 um)

To digitize our slides we used a customized Olympus BX60 microscope equipped with a LUDL's BioPrecision motorized stage, a high speed tiling system (Objective Imaging), and a Retiga 2000R digital color camera. The histology slides were scanned using a 40× Olympus UPlanApo objective (400× magnification) lens, allowing for the visualization of the cell nuclei. A Dual-Core Intel Xeon 3.0GHz, 2GB Ram, Dell Precision workstation was used for the analysis. For automated image processing and analysis, the histology slide images were partitioned into 64 (8 by 8) *sections *of size 1150 by 1540 pixels and then each of these section-images was partitioned again into 25 (5 by 5) sub-images (Fig 1). As a result, the *basic input *in our low-level image processing steps was a *sub-image *of size 230 by 310 pixels, with 1 pixel corresponding to 0.73 μm in our image system. In cell-crowded regions, sub-images contained hundreds of cell nuclei. The resulting image dataset contained 38,400 sub-images.

### Morphology classification of cell nuclei on histology slides and training and validation datasets

The tissue micro-textures in the images were classified as follows [18]:

#### NM1 (Nucleus Morphology 1)

Dark round spots (small dark blue nuclei with no chromatin clearing) typically corresponding to the nuclei of inflammatory cells (lymphocytes, plasma cells, macrophages and mast cells) circulating in blood vessels or invading the surrounding tissue.

#### NM2

Nuclei of cells of epithelial origin having nearly uniform chromatin distribution. These nuclei were significantly larger than the nuclei of lymphocytes.

#### NM3

Nuclei of cancer cells with irregular (non-uniform) chromatin distribution [9]. These nuclei assumed extensive shape variation and had chromatin clearing and prominent pleomorphic nucleoli.

#### ECM (Extra Cellular Matrix)

The collagen-based matrix supporting the cells in the stroma.

#### AT (Adipose Tissue)

Areas representing water, carbohydrate, lipid or gas.

**Figure 2:**
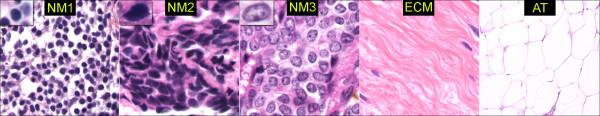
**Tissue microtextures identified using image processing**. The first three columns show examples of cell nuclei belonging to nucleus morphology categories NM1, NM2, and NM3, respectively. The textures identified as ECM and AT represent, respectively the collagen-rich stroma, and fat and tissue-devoid regions of the slide.

Examples of these micro-textures are shown in Fig 2.

### Automated image analysis

**Figure 3:**
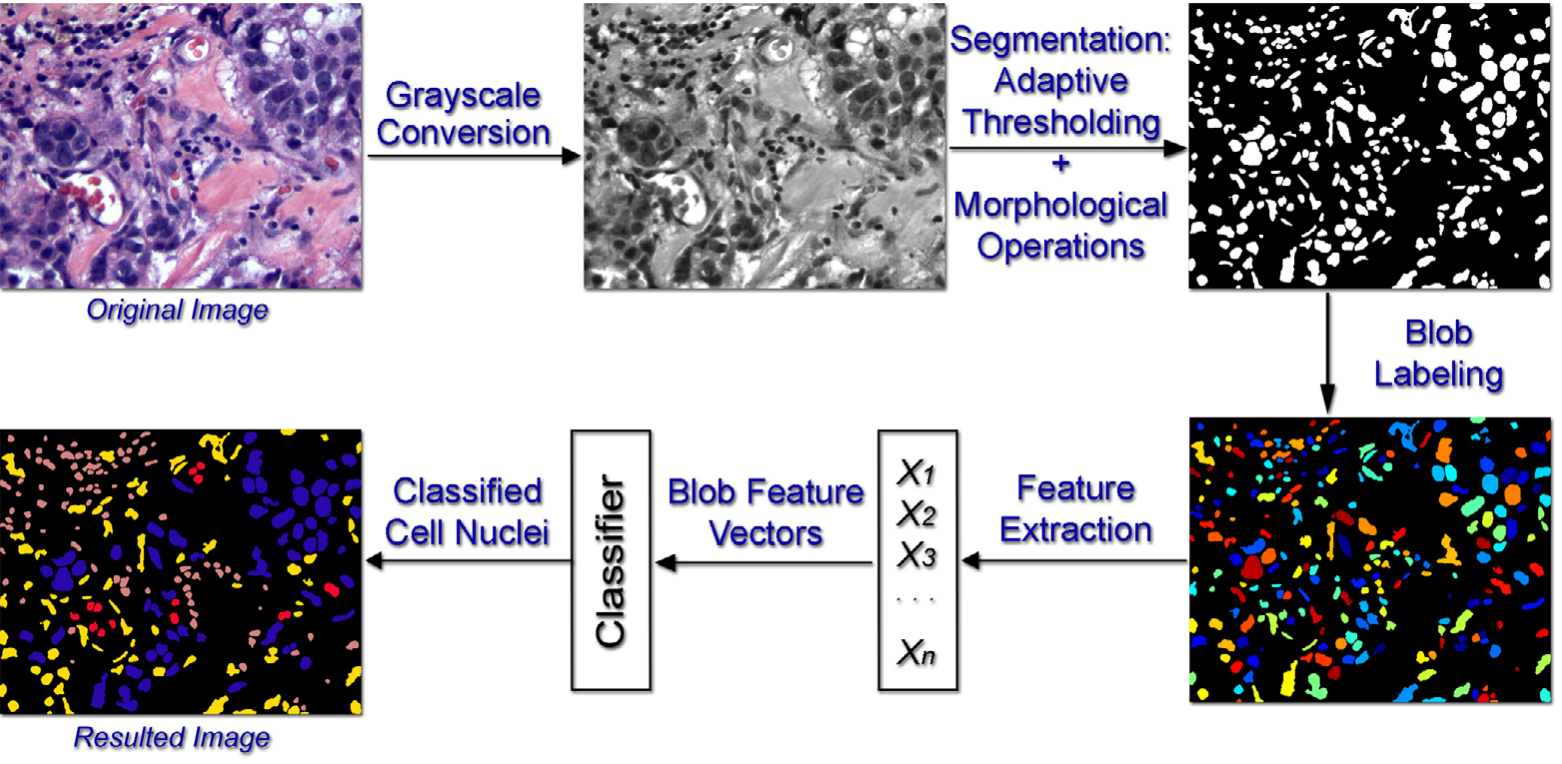
**Flowchart for sub-image level automated image processing**. The steps involved include greyscale conversion, segmentation with adaptive thresholding and morphological operations, blob (micro-object) labelling, feature extraction, blob classification, and nuclei classification.

Our method for the automated image processing of 38,400 sub-images was previously described by Petushi et al. [18]. MATLAB Image Processing Toolbox. was used for prototyping of the algorithms designed for this study. Similarly, the graphical user interface used to automate the image processing/analysis in this study was designed in the MATLAB programming environment. The process included grayscale conversion, segmentation with adaptive thresholding and morphological operations, blob (micro-object) labelling, feature extraction, blob classification, and nuclei classification using supervised learning (Fig 3). *Training and validation datasets *for the identification of cell nuclei of different morphology types using supervised learning were generated from the histology images used in this research. Both datasets contained 250 examples of cell nuclei in three morphology categories and 250 blobs that were deemed visually as artefacts and not nuclei. Identification of the micro-textures shown in Figure 2 on the histology images used in this study allowed us to determine surface number density of nuclei and detect the presence of higher order structural objects in the images such as cross sections of breast ducts as closed regions of high pixel intensity that are surrounded by cell nuclei.

### High nuclei density regions

**Figure 4:**
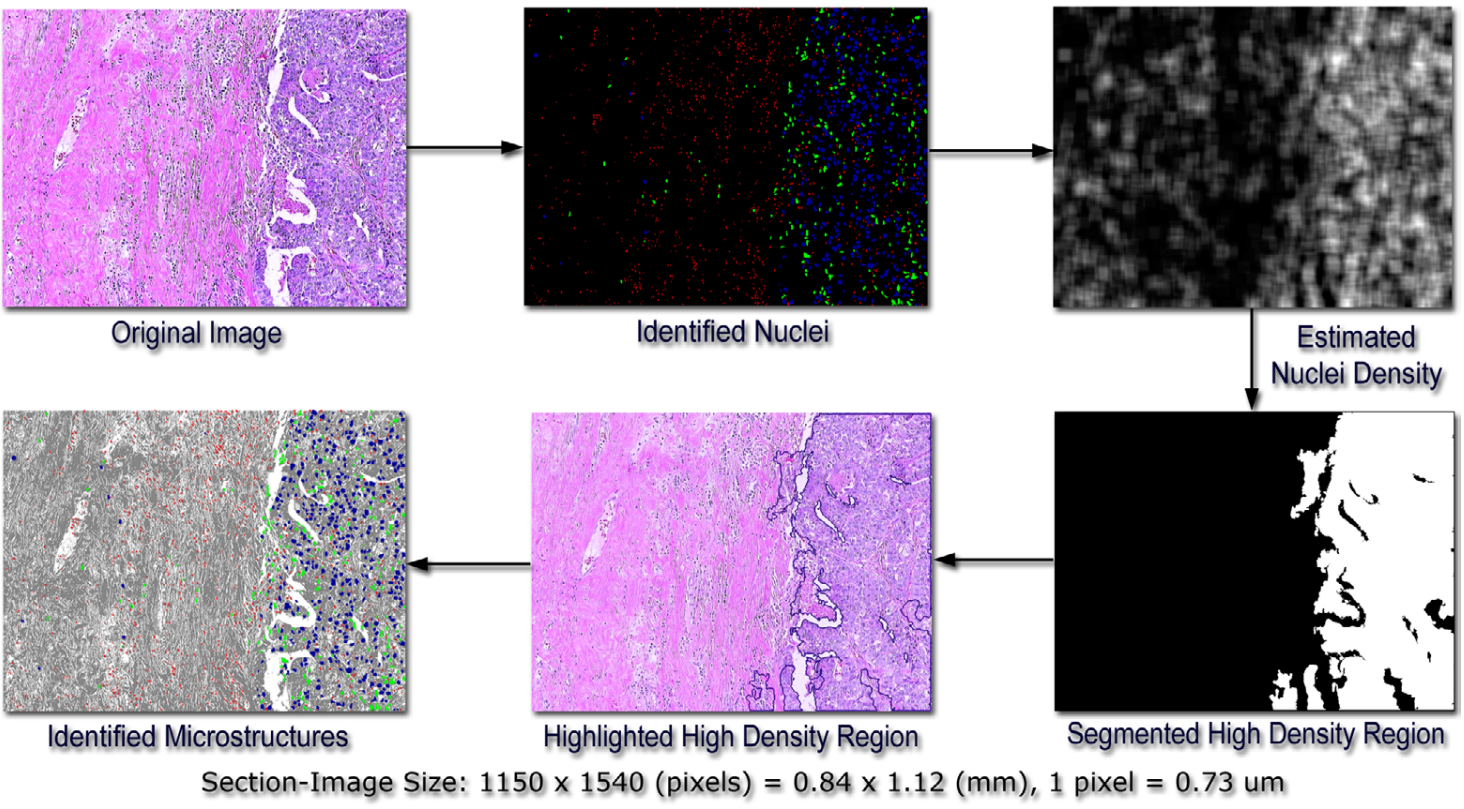
**Flowchart for section-image level processing**. Original image is processed to identify the spatial positions of thousands of cell nuclei in each section image. This information is then used to compute nuclei density at each pixel of the section image using a square window (50 pixels × 50 pixels). The resulting histogram undergoes adaptive segmentation to identify regional maxima indicating high-nuclei density regions.

High nuclei density regions in each section image were identified as follows: a square window, large enough to contain multiple nuclei (50 pixels × 50 pixels), scanned whole section-images and assigned a density value for the NM1, NM2, and NM3 type nuclei (number of nuclei pixels per square image window) to each pixel. The result of this filtering operatorion was the generation of a topological like map with the regional maxima indicating high nuclei distributions for each nuclei type (NM1, NM2, NM3, NM1–NM2, NM1–NM3, NM2–NM3, NM1-NM2-NM3). These nuclei density section-images were then segmented to identify the regional maxima representing high-density concentration of nuclei. Segmentation was composed of adaptive optimal thresholding and standard morphological filling and edge smoothing operations [19,20] (Fig 4). If the section-image did not posses more than 300 identified nuclei, it was rejected from further processing in order to assure statistically significant nuclei density values for each processed section image. The whole slide images that did not contain at least 11 section images having a minimum of 300 nuclei were also eliminated from the dataset. There were two such slide images in the Grade I category and one in the Grade II category. The remaining slide images in the set contained a minimum of 29 section images out of the maximum 64. Note that a slide image is composed of 64 section images. The filtering process reduced the number of section images in the study from 24 × 64 = 1536 to 1062.

Given that a section-image has passed filtering based on at least 300 identified nuclei, numbers of NM1, NM2, and NM3 nuclei per unit high nuclei density area were calculated for each section image and denoted as DNM1, DNM2, DNM3, DNM1-2, DNM1-3, DNM2-3, DNM1-2-3 respectively.

### Other texture features

**Figure 5:**
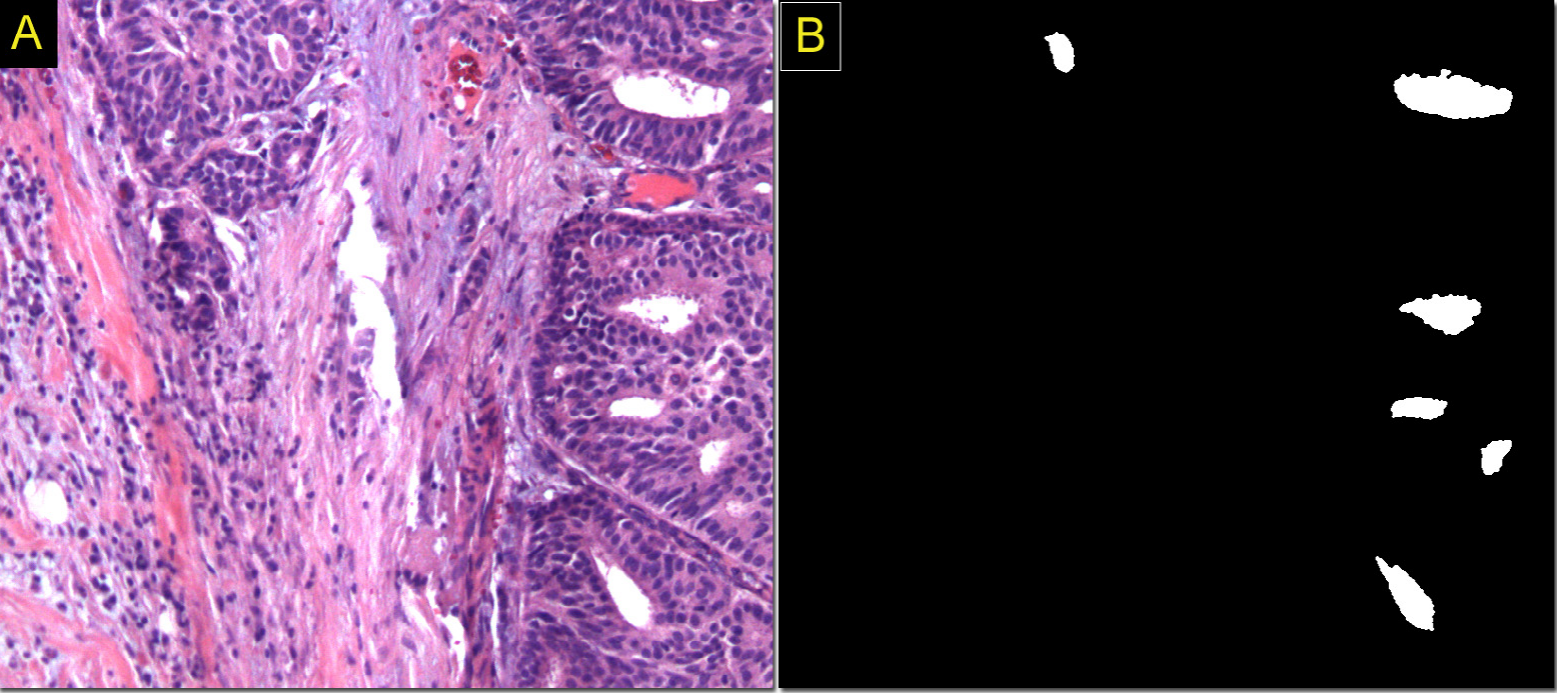
**Breast tubular cross-sections identified as white regions surrounded by cell nuclei**. Original section-image is shown in 5A, whereas the image indicating the tubules identified by our software is presented in 5B.

The number of tubules (DT) per unit high nuclei density area was determined by identifying high pixel intensity regions that were surrounded by a string of cell nuclei (Fig 5). The average distance (DN) between the centroids of closest nuclei in the high-density regions was also determined for each section image as a possible grade- differentiating feature. Therefore, the feature vector for each section image contained 9 features: DNM1, DNM2, DNM3, DNM1-2, DNM1-3, DNM2-3, DNM1-2-3, DT, and DN.

### Classification of section images using guidance from assigned grade

We used the LNKnet [21] software package in order to classify the section images using guidance from grade in a training and validation dataset. LNKnet is a software package that integrates neural network, statistical, and machine learning classification, clustering, and feature selection algorithms into a modular software package.

**Figure 6:**
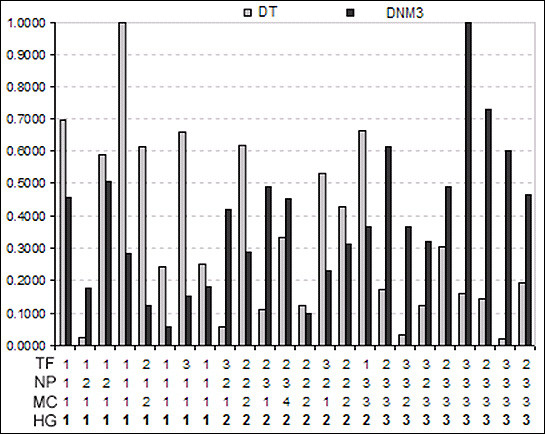
**Normalized densities of the two selected grade-differentiating features in relation to Nottingham scores and histologic grade of pathology evaluation**. Bar graphs indicating the values of tubular cross section density (DT) and density of nuclei morphology 3 (DNM3) for each of the 24 slides used in the study. Also shown in the figure for each slide are the Nottingham scores: tubular formation (TF), nuclear pleomorphism (NP), mitotic count (MC), and hitologic grade (HG).

**Figure 7:**
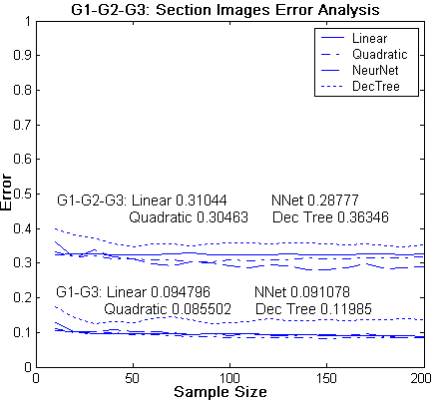
**Classification error analysis of the four classifiers in the LNKNET (linear, quadratic, neural network, and decision tree) in our testing data**. Classifiers were trained based on the two selected grade-differentiating features (DNM3 and DT) and their correlation to Histologic grade assigned by the two pathologists.

The section image dataset of 1062 feature vectors were divided into two: one containing 536 feature vectors for training and the other containing 526 feature vectors for testing by equally portioning section images of each slide into these subsets. If the number of section images of a slide was an odd number, the number of section images assigned to training from this slide was one greater than the number of section images assigned to the testing set. Going through the process of feature selection, using both Linear Discriminant Analysis (LDA) and Forward/Backward Search methods in LNKnet, two of the extracted higher order features, DNM3 and DT, were identified as accurately predicting the histologic grade significantly more frequently than the other seven. These two discriminant inputs were the DNM3 and the DT. Figure 6 shows the whole-slide normalized densities of these two features in all 24 patient cases in relation to the Nottingham scores and Histologic grade assigned by the two pathologists. Using these two Discriminant features different supervised classifiers in the LNKNET were trained and tested. Out of various available classifiers (linear, quadratic, neural network, decision tree, etc.) in the LNKnet, the quadratic classifier resulted in providing minimum mean classification errors when evaluated in the testing set (Fig. 7).

## Results and discussion

In this study, we have used an automated imaging algorithm that identifies cancer cell nuclei in histology slide images and classifies these nuclei into three categories according to morphology [18]. The algorithm also determines the spatial patterns in cell nuclei distribution on histology images. The micro-texture parameters obtained from histology images are potential indicators of cancer type and grade. The end result of the image processing on histology slides was the separation of the image into the following regions: adipose and tissue-empty regions of the slide (AT), ECM, and the cell nuclei classified into three categories according the morphology (NM1, NM2, NM3). In this way, automated procedure identified the spatial positions of hundreds of thousands of cell nuclei per histology slide image.

**Figure 8:**
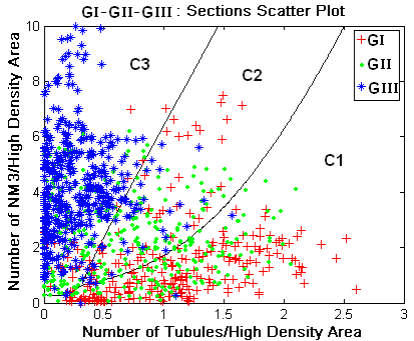
**Bar graphs depicting the level of agreement on nuclei morphology grading by visual inspection and automated image processing**. Each bar indicates the results of our automated classification of microtextures, previously identified by pathologist as NM1, NM2, NM3 cell nuclei and artefacts (250/class).

Computations with the labelled dataset of image objects showed close match on nuclei identification by visual inspection and automated image processing. Bar graphs in Figure 8 shows the classification results from the testing dataset that were obtained by using the LNKnet binary decision tree classifier composed of 50 nodes and 51 leaves. The results depicted by the bar graphs in this figure indicate that the automated procedure described in this study correctly identified blobs caused by stains and other artefacts as non-nuclei 239 out 250 times (95.6%) and assessed only 24 cell nuclei out of 750 (3.2%) as artefacts (not nuclei). Thus the automated system used in the study identified 4.4% of objects that pathologists considered as artefacts as cell nuclei and 3.2% of the objects that pathologists considered as nuclei as artefacts. These results indicate that automated image processing predictions are in line with those of visual inspection and compare well with results of other studies [12-14].

The discrepancy between automated predictions of nuclear morphology type and visual assessment are also shown in Figure 8. The procedure identified about 20 % of nuclei deemed as NM3 by visual inspection as NM2 nuclei. In the set of 250 nuclei previously labelled by visual inspection as belonging to the NM3 morphology, our automated system identified 176 nuclei as NM3 nuclei, 64 as NM2 nuclei and 10 as ECM. No nuclei previously graded as NM1 were identified by automated imaging as NM3 whereas only a small percentage of NM2 graded nuclei were identified by our automated system as NM3 (7/250). These results indicate that NM3 could be underestimated in some slides by as much as 20% compared to visual evaluation. But since all slides were evaluated using the same automated image analysis the results could be expected to be consistent within the dataset.

### Classification of section images

**Figure 9:**
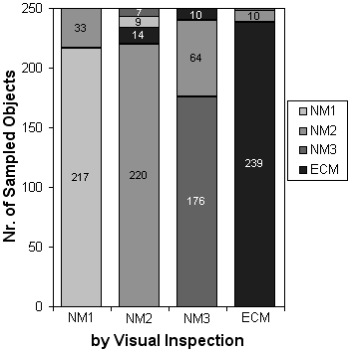
**Scatter plot for the classification of section images using DNM3 and DT as feature parameters**. Continuous lines identify the boundaries of section image classes C1, C2, and C3 as computed by using a quadratic classifier.

**Figure 10:**
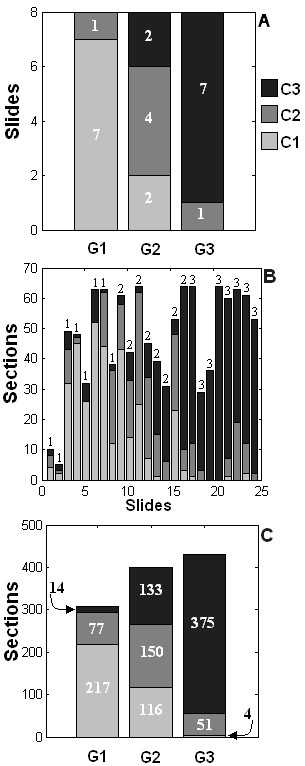
**Bar graphs indicating the numbers of section images in C1, C2, and C3 categories of each histology slide used in the study**. The symbols GI, GII, and GIII refer to the tumor grades associated with each slide.

Pathologists frequently use subsections of the image of a whole section histology image. Therefore, we focused on the classification of 1062 section images derived from the 24 whole section slide images. The feature vector for each section image consisted of the number density of nuclei (DNM1, DNM2, DNM3, DNM1-2, DNM1-3, DNM2-3, DNM1-2-3), the number density of tubules (DT) and the mean distance between neighbouring nuclei (DN). As described in the methods section, supervised classification was used to identify the discriminant features out of the nine features previously described. The classification indicated DNM3 and DT as grade differentiating parameters. The scattering diagram shown in Figure 9 identifies DNM3 and DT values for each of the section images used in the study. Section images are identified as point symbols in this graph by the grade of the whole slide to which they belong. The figure shows that typically DNM3 increased and DT decreased with increasing grade of histology images but that the feature coordinates of section images did not group into three easily identifiable clusters. We then used a quadratic classifier from the LNKnet software package to allocate the section images into three classes identified as C1, C2, and C3. As described in the Methods section, the quadratic classifier was trained to associate class 1 (C1) of section images with Grade I (GI), C2 with GII and C3 with G3. The continuous lines that separate the scatter plot in Figure 9 into three regions identify the boundaries of the C1, C2, and C3 classes of section images that were determined by the quadratic classifier. The figure shows that the section images previously graded by pathologists as grade I and III (GI, GIII) were well separated in classes C1 and C3 whereas half of the slide-images graded as II (GII) were allocated into C2 and the rest into C1 or C3. This point is further emphasized in Figure 10 in the bar graphs indicating the number of C1, C2, and C3 category section images in each histology slide in the data set. The figure shows that the grade of the tumor is strongly correlated with the most frequently observed classification type in the section images of the slide.

Automated image processing techniques such as ours have the capability to scan through whole section images and identify regions of similar texture, represented in this case by C1, C2, and C3 classification. The bar graphs shown in Figure 10 further indicates the potential to exploit tumor tissue image heterogeneity to identify subclasses associated with each tumor grade. This sub-classification could depend on the C1, C2, and C3 composition of histology slides. Further studies are needed to explore such ideas in more detail.

## Conclusion

This article presents an automated classification of breast histology images using two texture features: surface density of nuclei and subsequently spatial position. Surface density is assessed by a technique that detects and identifies millions of cell nuclei on the images of histology slides and classifies them into three categories according to morphology [18]. The spatial positions of cell nuclei are then used to detect higher order tissue structures such as tubular cross sections and the boundaries of high nuclei density regions. The program developed enables a systematic investigation of image micro-texture parameters with respect to their potential to differentiate stage and type of breast cancer.

Our study focused on the images of H & E stained histology slides because this stain is the most widely used in diagnostic pathology [1]. H & E stains DNA blue and collagen pink and hence allows segmentation of the cell nuclei from the stroma. Image analysis of H&E staining is a challenging task due to technical and tumor factors such as the wide variations found in the shade and intensity of staining and the non-uniform chromatin distribution in cancer cell nuclei. Despite these difficulties, our automated adaptive segmentation method enabled us to distinguish the adipose and/or tissue-void spaces from the adjoining stroma. Adaptive segmentation was also quite effective in identifying cell nuclei with nearly uniform pixel intensity. However, supervised learning and subsequent classification had to be utilized to distinguish those cell nuclei with highly variable chromatin stain intensity from the surrounding tissue.

Our computations identified cell nuclei in three different morphologic categories. Nuclei in NM1 category typically had uniform pixel intensity and represented the nuclei of lymphocytes and other immune cells. Nuclei in NM2 had higher pixel intensity and a larger size compared to NM1. The nuclei in this category were found in all three grades of cancer. NM3 nuclei, on the other hand, displayed wide variation of chromatin distribution and were found preferentially in high-grade cancers. Automated detection of these tissue micro-objects on the histologic image not only eliminated the bias of the human eye in grading histology slides but also enabled us to detect and identify the nuclei type of hundreds of thousands of cells per slide, a task that could not be accomplished by visual determination of nuclei morphology.

The high nuclei-density regions of the stroma were computed next using the adaptive segmentation method described in the Materials and Methods section. These regions potentially contain significant information about the progression of the tumor. We found the extent of fat tissue and tissue-devoid regions of the image varied sharply from slide to slide even in the subsets of identically graded histology slides and therefore this parameter could not be used in clinically relevant classification of the histologic images. On the other hand, the spatial distribution of nuclei data permitted the identification of breast ducts and associated tubular sections on the histology slides. Distinction of tumor section images using various classification algorithms based on supervised learning attested to the grade differentiating capacity of two micro-object features: the number of NM3 nuclei per unit area (DNM3) and the number of tubules per unit area (DT) in the high nuclei density region of the stroma. Classification of section images using these two texture parameters separated images obtained from Grade I and Grade III histology slides into distinct categories (C1, C3) and placed the images obtained from Grade II slides in between these two categories. In many instances section images obtained from the same slide were classified into different categories (C1, C2, C3) but the grade of the slide (GI, GII, GIII) was typically associated with the most frequently observed classification type. We forsee incorporation of additional features such as the number density of dividing nuclei and the intensity of stains identifying cancer associated molecular markers into the classification scheme to achieve a comprehensive sub classification of breast tumors.

Our results suggest that computer-based image processing and analysis can be used in to assist pathologists performing this task and lead to a reduction in intra-observer variability. The clinical potential of the image processing and classification scheme presented here will increase when studied in combination with other features from multiple cancer evaluation platforms such as biomarker-decorated image data, global gene expression profiles and chromosome aberration measurements.

## Competing interests

The author(s) declare that they have no competing interests.

## Authors' contributions

SP carried out the acquisition and digitization of histology slides, designed and implemented the algorithms for the image processing, analysis and classification of the histology slides, and helped to draft the manuscript. FG and MH carried out the pathologic grading and selection of histology slides and guided SP in generating the training and validation datasets. CK participated in the implementation of image processing and analysis algorithms. AT supervised and coordinated the entire project, participated in the analysis process, and helped to draft the manuscript. All authors read and approved the final manuscript.

## Pre-publication history

The pre-publication history for this paper can be accessed here:


